# Investigation of correlation between shear wave elastography and lymphangiogenesis in invasive breast cancer and diagnosis of axillary lymph node metastasis

**DOI:** 10.1186/s12885-024-12115-x

**Published:** 2024-04-02

**Authors:** Bo Li, Shaochun Dai, Qiucheng Wang, Hui Jing, Hua Shao, Lei Zhang, Ling Qin, Cong Qiao, Zhuozhong Wang, Wen Cheng

**Affiliations:** 1 Department of Ultrasound, Harbin Medical University Cancer Hospital, 150 Haping Rd, Nangang District, 150081 Harbin, China; 2 Department of Pathology, Harbin Medical University Cancer Hospital, Harbin, China; 3 Department of Pathology, Harbin Medical University, Harbin, China; 4 The Key Laboratory of Myocardial Ischemia, Harbin Medical University, Ministry of Education, Harbin, China

**Keywords:** Shear wave elastography, Lymphangiogenesis, Lymph node metastasis

## Abstract

**Background:**

Accurate evaluation of axillary lymph node metastasis (LNM) in breast cancer is very important. A large number of hyperplastic and dilated lymphangiogenesis cases can usually be found in the pericancerous tissue of breast cancer to promote the occurrence of tumor metastasis.Shear wave elastography (SWE) can be used as an important means for evaluating pericancerous stiffness. We determined the stiffness of the pericancerous by SWE to diagnose LNM and lymphangiogenesis in invasive breast cancer (IBC).

**Methods:**

Patients with clinical T1-T2 stage IBC who received surgical treatment in our hospital from June 2020 to December 2020 were retrospectively enrolled. A total of 299 patients were eventually included in the preliminary study, which included an investigation of clinicopathological features, ultrasonic characteristics, and SWE parameters. Multivariable logistic regression analysis was used to establish diagnostic model and evaluated its diagnostic performance of LNM. The correlation among SWE values, collagen volume fraction (CVF), and microlymphatic density (MLD) in primary breast cancer lesions was analyzed in another 97 patients.

**Results:**

The logistic regression model is Logit(P)=-1.878 + 0.992*LVI-2.010*posterior feature enhancement + 1.230*posterior feature shadowing + 0.102*posterior feature combined pattern + 0.009*Emax. The optimum cutoff value of the logistic regression model was 0.365, and the AUC (95% CI) was 0.697 (0.636–0.758); the sensitivity (70.7 vs. 54.3), positive predictive value (PPV) (54.0 vs. 50.8), negative predictive value (NPV) (76.9 vs. 69.7), and accuracy (65.2 vs. 61.9) were all higher than Emax. There was no correlation between the SWE parameters and MLD in primary breast cancer lesions.

**Conclusions:**

The logistic regression model can help us to determine LNM, thus providing more imaging basis for the selection of preoperative treatment. The SWE parameter of the primary breast cancer lesion cannot reflect the peritumoral lymphangiogenesis, and we still need to find a new ultrasonic imaging method.

## Background

Breast cancer is the most common malignant tumor in Chinese women. Metastasis is the leading cause of incurable and fatal diseases in patients who suffer from breast cancer. Lymph node metastasis (LNM) can occur at an early stage. Preoperative assessment of LNM is necessary, as it can guide the clinical staging classification of breast cancer and the selection of treatment options [1]. Axillary lymph node dissection (ALND) is required in patients with LNM and may lead to complications such as lymphedema, limited movement of the arms and shoulders, and numbness of the upper arm skin [2]. US-guided fine-needle aspiration is an important nonsurgical alternative to ALND, but it remains an invasive procedure that inevitably produces false-negative results [3]. Therefore, it is necessary to explore a noninvasive method for the preoperative evaluation of axillary lymph node.

As a complement to conventional ultrasound and color doppler flow imaging, shear wave elastography (SWE) can display a real-time color map to determine the stiffness of the mass by observing the color features, and quantitatively measure the values by subsequently placing a region of interest (ROI) on the hardest part of the lesion. According to a multicenter study on E-Imaging of the Breast in China (BE3) in 2016, which was cohosted by Professor Chang and Professor Li, breast cancer usually shows a stiff rim, colored lesion, defect filling, or horseshoe sign, which has diagnostic value. SWE can be used as an important means for evaluating pericancerous stiffness which may assist the diagnosis of LNM.

Lymphangiogenesis is an important condition for the LNM of tumors. There is a correlation between survival rate and lymphatic vessel density (LVD) in patients with different types of tumors. LNM can occur by the invasion of the existing lymphatic vessels located within [4, 5] or surrounding [6] the tumor and tumor-induced lymphangiogenesis. A large number of hyperplastic and dilated lymphangiogenesis cases can usually be found in the pericancerous tissue of breast cancer to promote the occurrence of tumor metastasis. VEGFC plays a key role in the lymphangiogenesis and LNM of tumors, and TGF-β can stimulate lymphangiogenesis by promoting the expression of VEGFC. The transformation of breast cancer is accompanied by an increase in collagen deposition and a progressive linearization and thickening of interstitial collagen, which is most significant in pericancerous tissue. There is a positive correlation between stromal stiffness and the level of cellular TGF-β signaling [7]. The main contributor to tumor stiffness is collagen-rich extracellular matrix (ECM) [8]. Lesion stiffness is closely related to collagen content, the proteins that promote tissue fibrosis and collagen expression [9]. Therefore, whether SWE is related to lymphangiogenesis is a feasible research direction, that may help to evaluate the therapeutic efficacy against tumor lymphangiogenesis.

## Methods

### Ethical statement

The research was approved by the ethical review board of Harbin medical university cancer hospital, and was consistent with the Helsinki Declaration of 1964 and its corresponding revision or equivalent ethical principles. The informed consent of each patient was waived due to its retrospective nature by the ethical review board of Harbin medical university cancer hospital. Information that may reveal the identity of the recipient was exempted.

### Patients

Patients with invasive breast cancer (IBC) in the clinical T1-T2 stage who received surgical treatment in our hospital from June 2020 to December 2020 were retrospectively enrolled. A total of 299 patients were eventually included in the preliminary study, which included an investigation of clinicopathological features, ultrasonic characteristics, and SWE parameters. Multivariable logistic regression analysis was used to establish diagnostic model and evaluated its diagnostic performance of LNM. In patients with multifocal cancer, only the largest tumor was recorded. The exclusion criteria were as follows: patients receiving neoadjuvant chemotherapy (NAC); patients whose lesions had been excised or subjected to vacuum-assisted biopsy; patients who underwent biopsy prior to ultrasound or SWE examination; and patients with bilateral breast cancer. According to the presence of LNMs in patients after surgery, they were divided into LNM + or LNM- groups.

### Ultrasound and SWE examination

The ultrasound and SWE images were collected and stored using the Supersonic Imagine ultrasound system (Aixplorer, Aixen Provence, France) with a 2–10 MHz or 4–15 MHz probe. The work was performed by two experienced operators who had worked for more than 10 years. The ultrasonic characteristics and SWE parameters were independently evaluated by two experts. If there were any disagreements during the evaluation, a final conclusion was reached through consultation with another expert.

Primary breast lesions were recorded according to the 5th American College of Radiology Breast Imaging Reporting and Data System (BI-RADS) lexicon, including: shape, orientation, margin, echo pattern, posterior features, calcifications, and vascularity. Tumor size, halo, and distance from tumor to skin were also recorded.

For SWE, the ROI needs to include the whole mass and normal tissues around the mass. The Q-box diameter was set to 2 mm. The Q-box was placed in the hardest part of the peritumoral lesions. When measuring the Eratio, the second Q-box is placed in the normal adipose tissue with the same depth as the lump as a reference. (If the lump is too large, it can be placed in the normal mammary gland with the same depth.) SWE images perpendicular to each other were stored, and maximum stiffness (Emax) (kPa), Mean stiffness (Emean) (kPa), Stiffness ratio (Eratio) and Stiffness standard deviation (Esd) (kPa) in each image were recorded.

### Histopathological evaluation

Estrogen receptor (ER), progesterone receptor (PR), human epidermal growth factor receptor 2 (Her2), and Ki-67 were detected by immunohistochemistry. ER and PR states were positive when more than 1% of the tumor cells were immunohistochemically stained [10]. HER2 positivity was defined as grade 3+, and grade 2 + needed to be detected by fluorescence in situ hybridization. Greater than 20% of Ki-67 was considered to be the threshold for the classification of the luminal subtype.

### Microlymphatic density

MLD was taken to evaluate lymphangiogenesis. An optical microscope (Leica, Germany) was used to observe the distribution areas of pericancerous positive staining under low magnification (10 × 4) to select hot spots; three hot spots were selected for each section. Under high magnification (10 × 10), one image was collected from each hot spot. According to the Weidner lymphatic vessel counting method [11], the number of microlymphatic vessels peritumorally stained tan by D2-40 antibody in each image was manually counted. Any tan-staining endothelial cell or endothelial cell cluster was considered a single countable microlymphatic vessel. The mean number of microlymphatic vessels in the three images per slice was taken as the final MLD value.

### Collagen

Masson staining was performed on collagen. After Masson trichrome staining, the collagen fibers were blue, the nuclei were blue–black, and the muscle fibers and red blood cells were red. An image acquisition system was used to collect three peritumoral images for each slice under a high magnification field (10 × 10). ImageJ software ( http://rsb.info.nih.gov/ij/ ) was used to analyze the CVF, the collagen-positive area as a percentage of the total area of the organization.

### Statistical analysis

Statistical analysis was performed using SPSS 20.0 software (SPSS Inc., Chicago, IL, USA). Bilateral test results (test level α = 0.05) showed a statistically significant difference when *P* < 0.05. Measurement data with a normal distribution are described as the mean ± SD, and the difference between two groups was analyzed by t test. If the measurement data did not conform to a normal distribution, they are described as the median (interquartile range), and the difference between two groups was examined by the Wilcoxon rank sum test. Enumeration data are described by n (%), and the distribution differences of related factors between different groups were analyzed by the chi-square test. Factors with statistically significant differences will be additionally incorporated into the multivariable logistic regression model. Following the stepwise selection of independent variables, the model will undergo optimization to obtain the united diagnostic model. Receiver operating characteristic (ROC) curves were used to analyze the diagnostic efficacy of different indicators for metastasis. Delong’s test was used to analyze whether there was statistically significant difference in the diagnostic ability of each indicator. Pearson correlation and Spearman rank correlation were used to analyze the correlations among SWE values, CVF, and MLD. The results with statistical significance are shown in scatter plots.

## Results

Based on the inclusion and exclusion criteria and postoperative pathology, 299 IBC patients were eventually included in the preliminary study, including 183 (61.2%) LNM- patients and 116 (38.8%) LNM + patients. Another 97 patients with IBC who met the criteria were selected, including 50 LNM- (51.5%) patients and 47 LNM+ (48.5%) patients. The correlations among SWE values, CVF, and MLD in primary breast cancer lesions were analyzed in 97 patients.

Table 1 shows the disparities in clinicopathological features among patients with LNM- and LNM+. The distribution difference of lymphovascular invasion (LVI) between the two groups was statistically significant. In the LNM + group, the proportion of LVI was 34.5%, which was higher than that in the LNM- group (16.9%).

**Table 1:** Disparities in clinicopathological features among patients with LNM- and LNM+

Characteristic	LNM- (*n* = 183)	LNM+ (*n* = 116)	t/χ^2^	*P*
Age (Mean ± SD, years)	53.72 ± 10.00	55.13 ± 9.99	-1.191	0.235
Age (n, %)			1.303	0.521
≤ 45	42 (23.0%)	21(18.1%)		
45–60	91(49.7%)	58 (50.0%)		
>60	50(27.3%)	37 (31.9%)		
Histologic type (n, %)			2.608	0.271
Invasive ductal	169 (92.3%)	111(95.7%)		
Invasive lobular	4(2.2%)	3 (2.6%)		
Others	10(5.5%)	2 (1.7%)		
ER (n, %)			0.176	0.675
Negative	40 (21.9%)	23(19.8%)		
Positive	143(78.1%)	93 (80.2%)		
PR (n, %)			0.663	0.416
Negative	52 (28.4%)	28(24.1%)		
Positive	131 (71.6%)	88 (75.9%)		
HER2 (n, %)			3.678	0.055
Negative	147 (80.3%)	82(70.7%)		
Positive	36 (19.7%)	34 (29.3%)		
Ki-67 (n, %)			3.026	0.082
< 20%	93 (50.8%)	47(40.5%)		
≥ 20%	90(49.2%)	69 (59.5%)		
P53 (n, %)			1.591	0.207
Negative	52 (28.4%)	41(35.3%)		
Positive	131 (71.6%)	75 (64.7%)		
Molecular type (n, %)			7.083	0.069
luminal A	69 (37.7%)	41(35.3%)		
luminal B	75(41.0%)	53 (45.7%)		
HER2	15 (8.2%)	16(13.8%)		
TNBC	24(13.1%)	6 (5.2%)		
LVI (n, %)			12.067	**0.001**
Absent	152(83.1%)	76(65.5%)		
Present	31(16.9%)	40 (34.5%)		

*TNBC* triple negative breast cancer, *LVI* lymphovascular invasion

Regarding the ultrasound characteristics, there were statistically significant differences in tumor diameter and posterior features between the two groups (Table 2). The tumor diameter in the LNM + group was 21.43 ± 8.78 mm, whereas that in the LNM- group was 19.28 ± 7.86 mm (*P* = 0.028). The posterior feature enhancement was 1.7% in the LNM + group and 12.0% in the LNM- group.

**Table 2:** Univariate analysis of the variations in ultrasonic characteristics between the LNM- group and the LNM + group

Characteristic	LNM- (*n* = 183)	LNM+ (*n* = 116)	t/χ^2^	*P*
Tumor diameter (Mean ± SD, mm)	19.28 ± 7.86	21.43 ± 8.78	-2.204	**0.028**
Tumor size (n, %)			2.912	0.088
T1	116 (63.4%)	62(53.4%)		
T2	67(36.6%)	54 (46.6%)		
Distance from tumor to skin (Mean ± SD, mm)	6.66 ± 3.11	6.60 ± 2.97	0.164	0.869
Shape (n, %)			Fisher	1.000^a^
Regular	1 (0.5%)	0(0.0%)		
Irregular	182(99.5%)	116 (100.0%)		
Orientation (n, %)			0.059	0.808
Parallel	169 (92.3%)	108(93.1%)		
Not parallel	14 (7.7%)	8 (6.9%)		
Margin (n, %)			8.575	0.073
Circumscribed	1 (0.5%)	0(0.0%)		
Indistinct	105(57.4%)	53 (45.7%)		
Angular	35(19.1%)	20 (17.2%)		
Microlobulated	21(11.5%)	17 (14.7%)		
Spiculated	21(11.5%)	26 (22.4%)		
Halo (n, %)			0.352	0.553
Absent	162(88.5%)	100(86.2%)		
Present	21(11.5%)	16 (13.8%)		
Echo pattern (n, %)			2.608	0.271
Heterogeneous	4 (2.2%)	3(2.6%)		
Hypoecho	175(95.6%)	113 (97.4%)		
Isoecho or Hyperecho	4 (2.2%)	0(0.0%)		
Posterior features (n, %)			12.104	**0.007**
No features	148 (80.9%)	100(86.2%)		
Enhancement	22(12.0%)	2 (1.7%)		
Shadowing	3 (1.6%)	5(4.3%)		
Combined pattern	10 (5.5%)	9(7.8%)		
Calcifications (n, %)			0.280	0.597
Absent	26(14.2%)	14(12.1%)		
Present	157(85.8%)	102 (87.9%)		
Vascularity (n, %)			0.175	0.916
Absent	8 (4.4%)	4(3.4%)		
Internal vascular	108(59.0%)	70 (60.3%)		
Marginal vascular	67(36.6%)	42 (36.2%)		

^a^indicates Fisher exact test results

**Fig. 1:**
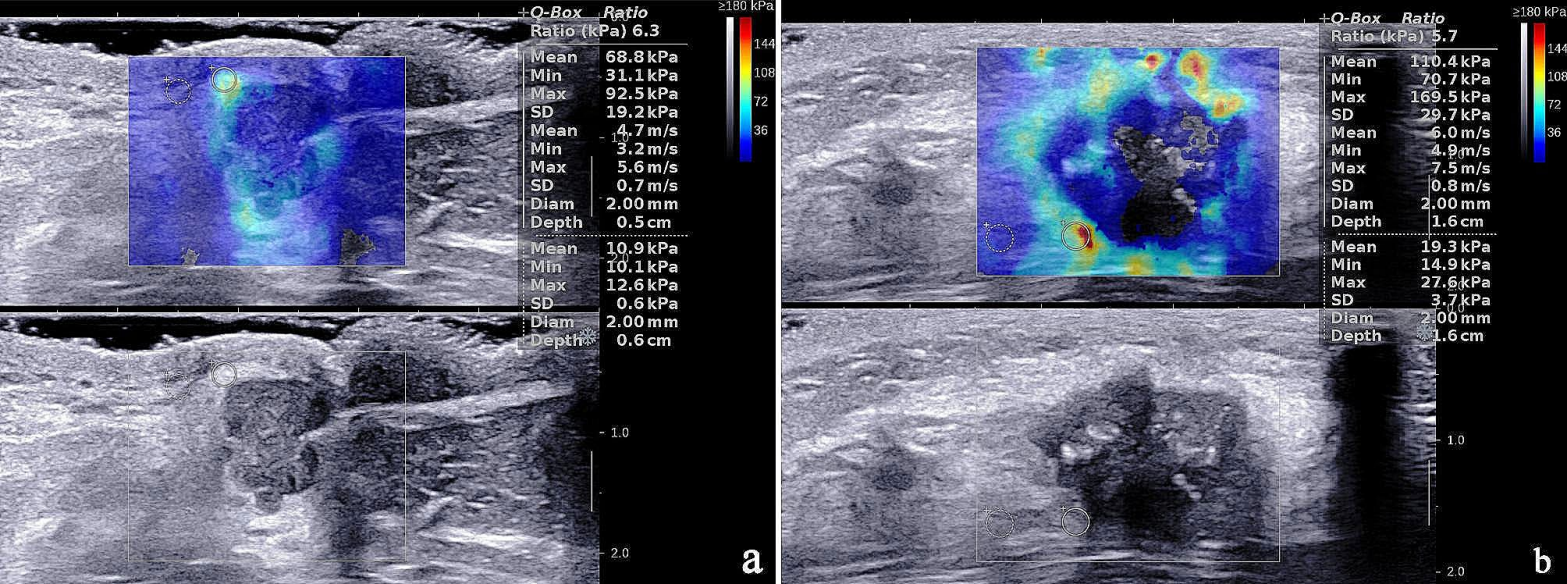
The SWE values of primary breast cancer in the LNM- (a) and LNM+ (b) groups. (a) SWE values of tumor were 92.5 kPa for Emax, 68.8 kPa for Emean, 6.3 for Eratio, 19.2 kPa for Esd. (b) SWE values of tumor were 169.5 kPa for Emax, 110.4 kPa for Emean, 5.7 for Eratio, 29.7 kPa for Esd

SWE examination of primary breast lesions showed that only Emax (146.73 ± 47.76 kPa vs. 132.07 ± 42.84 kPa, *P* = 0.006) had statistically significant differences in the distributions between the two groups (Table 3). Figure 1 shows the SWE values of primary breast cancer in the LNM- and LNM + groups.

**Table 3:** Univariate analysis of SWE parameters between the LNM- group and the LNM + group

SWE	LNM- (*n* = 183)	LNM+ (*n* = 116)	t/χ^2^	*P*
Emax (Mean ± SD, kPa)	132.07 ± 42.84	146.73 ± 47.76	-2.758	0.006
Emean (Mean ± SD, kPa)	109.46 ± 36.14	109.37 ± 34.29	0.021	0.983
Eratio (Mean ± SD, kPa)	11.18 ± 5.73	10.70 ± 4.80	0.754	0.451
Esd (Mean ± SD, kPa)	18.99 ± 8.89	18.99 ± 8.81	0.004	0.997

Factors with statistically significant differences in the distributions of different groups in univariate analysis (LVI, tumor diameter, posterior features, Emax) were further included in the multivariable logistic regression model. Following the stepwise selection of independent variables, tumor diameter was eliminated. The final logistic regression model was Logit(P)=-1.878 + 0.992*LVI-2.010*posterior feature enhancement + 1.230*posterior feature shadowing + 0.102*posterior feature combined pattern + 0.009*Emax.

As shown in Table 4, LVI (OR = 2.697, *P* = 0.001), posterior feature enhancement (OR = 0.134, *P* = 0.008), and Emax (OR = 1.009, *P* = 0.002) were independent predictors of LNM. Among them, tumors with posterior feature enhancement were less likely to have LNM, whereas tumors with LVI and larger Emax were more likely to have LNM.

**Table 4:** Multivariable logistic regression analysis for diagnosis of LNM + group

Variables	Estimate	SE	Wald χ^2^	OR (95%CI)	*p*-value
Intercept	-1.878	0.441	18.177	0.153(0.063,0.355)	< 0.001
LVI (Present vs. Absent)	0.992	0.291	11.658	2.697(1.533,4.804)	0.001
Posterior features (No features is the reference group)					
Enhancement	-2.010	0.757	7.045	0.134(0.021,0.478)	0.008
Shadowing	1.230	0.763	2.597	3.422(0.789,17.615)	0.107
Combined pattern	0.102	0.500	0.041	1.107(0.407,2.96)	0.839
Emax	0.009	0.003	9.334	1.009(1.003,1.015)	0.002

*LVI* lymphovascular invasion

**Fig. 2:**
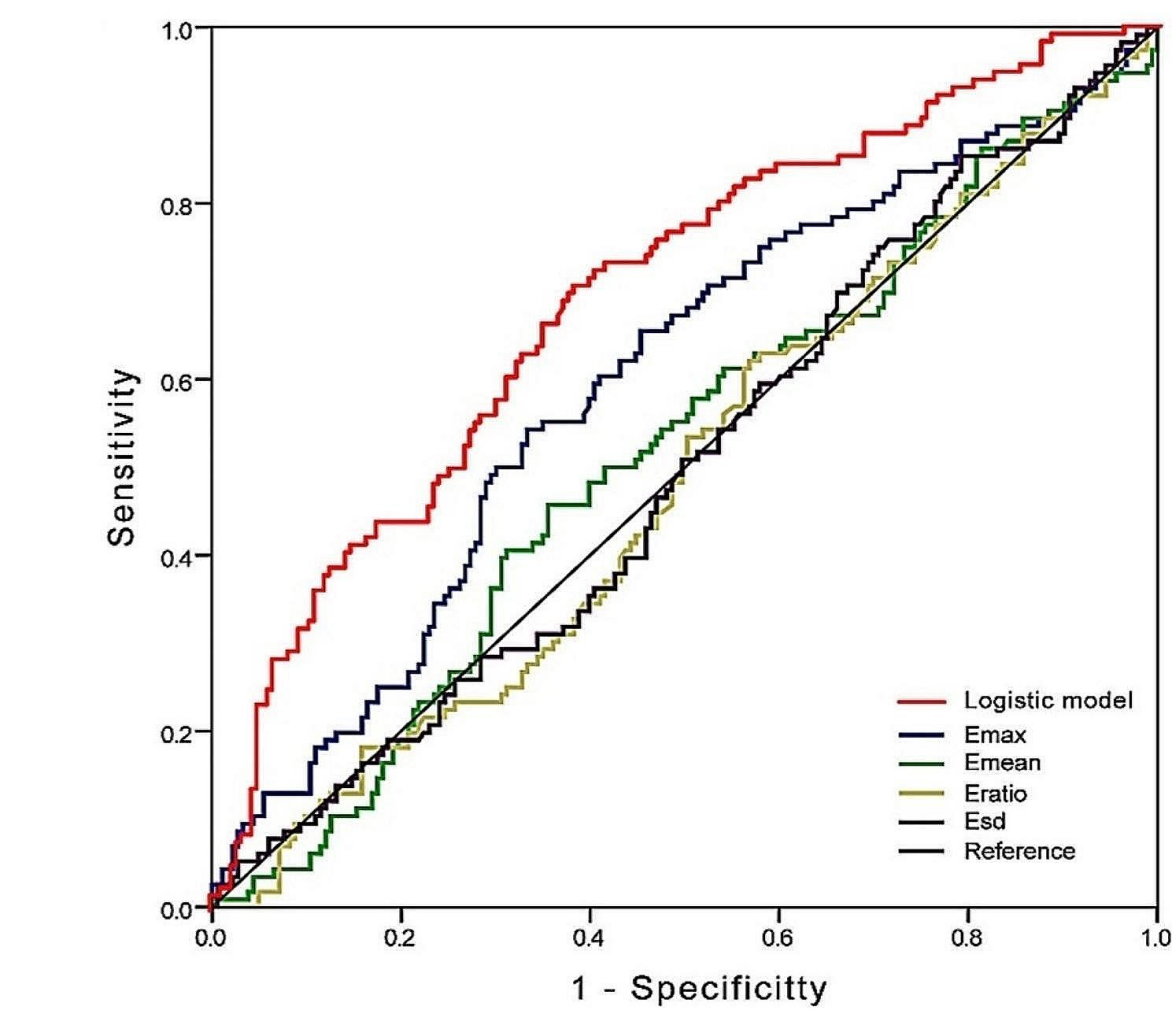
ROC curve for Emax, Emean, Eratio, Esd and logistic model to diagnosis LNM + of IBC. The logistic model has reliable application value (AUC = 0.697; 95% CI = 0.636–0.758). ROC receiver operating characteristic, IBC invasive breast cancer, AUC area under the curve, CI confidence interval

The ROC curves which are shown in Fig. 2 were used to analyze the diagnostic efficacy for metastasis. The logistic regression model had optimal diagnostic performance for metastatic outcome, and the differences between the logistic regression model and Emax, Emean, Eratio, and Esd were statistically significant (*P* < 0.05). In terms of the diagnostic efficacy of individual indicators, Emax had an optimal diagnostic performance for metastatic outcome, and the differences between Emax and both Eratio and Esd were statistically significant (*P* < 0.05), but the difference between Emax and Emean did not reach statistical significance. The optimum cutoff value of Emax was 144.455, and the area under the curve (AUC) (95% CI) was 0.600 (0.533, 0.666), whereas the optimum cutoff value of the logistic regression model was 0.365. The AUC (95% CI) was 0.697 (0.636–0.758), and the sensitivity (70.7 vs. 54.3), positive predictive value (PPV) (54.0 vs. 50.8), negative predictive value (NPV) (76.9 vs. 69.7), and accuracy (65.2 vs. 61.9) were all higher than those of Emax (Table 5). A logistic regression model cutoff 0.365, 71% of women with LNM would be detected, but if used for clinical decision making, 38% of women with LNM- disease would be potentially over treated.

**Table 5:** The diagnostic performance for the LNM + group with the optimal cutoff values

Variables	Cutoff value	AUC(95%CI)	Sensitivity(%)	Specificity (%)	PPV (%)	NPV (%)	Accuracy (%)
Emax	144.455	0.600 (0.533,0.666) ^a^	54.3	66.7	50.8	69.7	61.9
Emean	116.730	0.517 (0.450–0.584) ^ab^	45.7	64.5	44.9	65.2	57.2
Eratio	8.850	0.488 (0.421–0.555) ^b^	62.1	43.2	40.9	64.2	50.5
Esd	10.595	0.498 (0.431–0.565) ^b^	85.3	20.8	40.6	69.1	45.8
Logistic model	0.365	0.697 (0.636–0.758) ^c^	70.7	61.7	54.0	76.9	65.2

^abc^ Stands for Delong’s test, and there is no statistically significant difference in AUC between the same labeled indexes

**Fig. 3:**
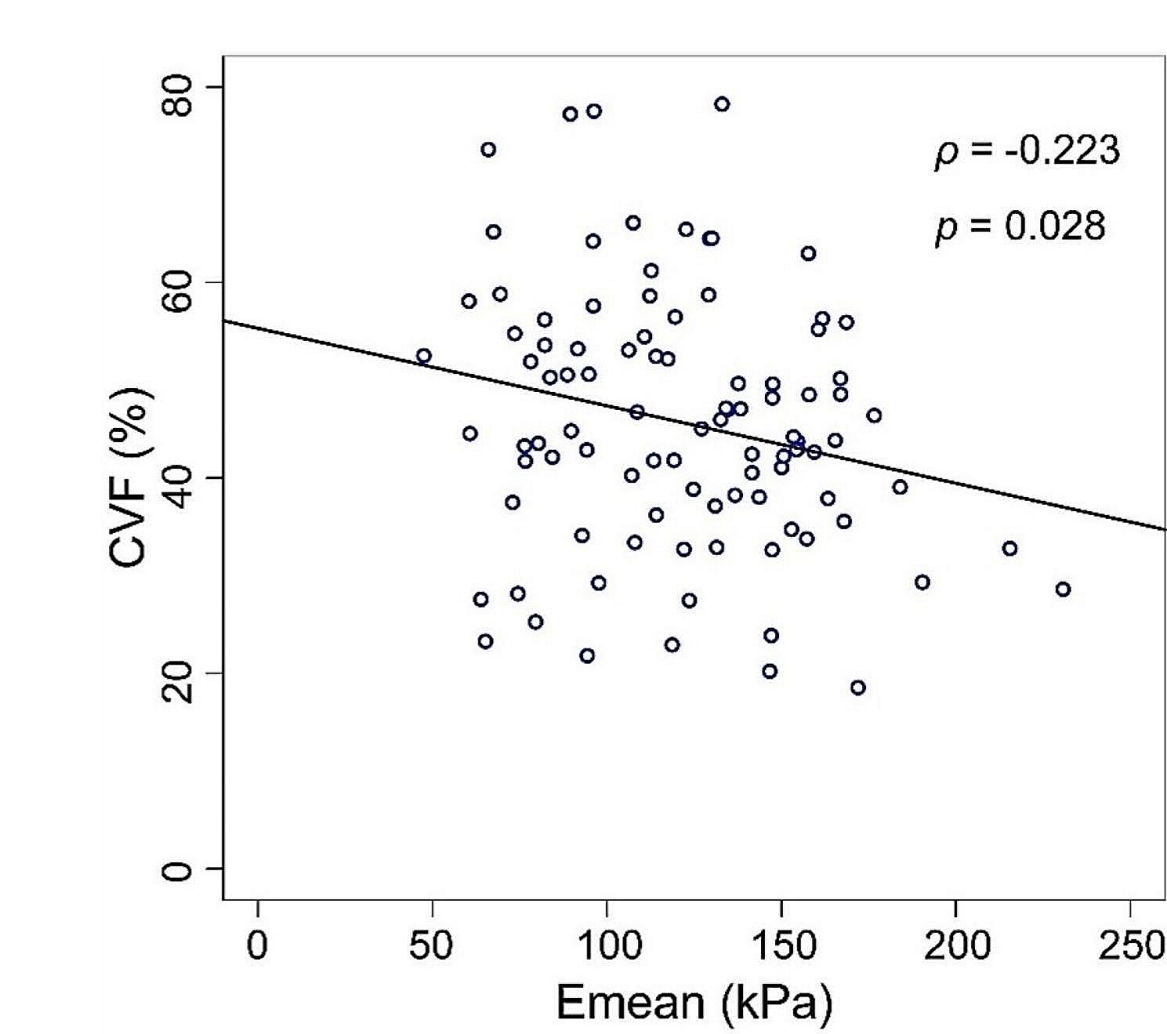
The scatter plot showed a weak negative correlation between CVF and Emean (ρ = − 0.223, *p* = 0.028). CVF collagen volume fraction

**Fig. 4:**
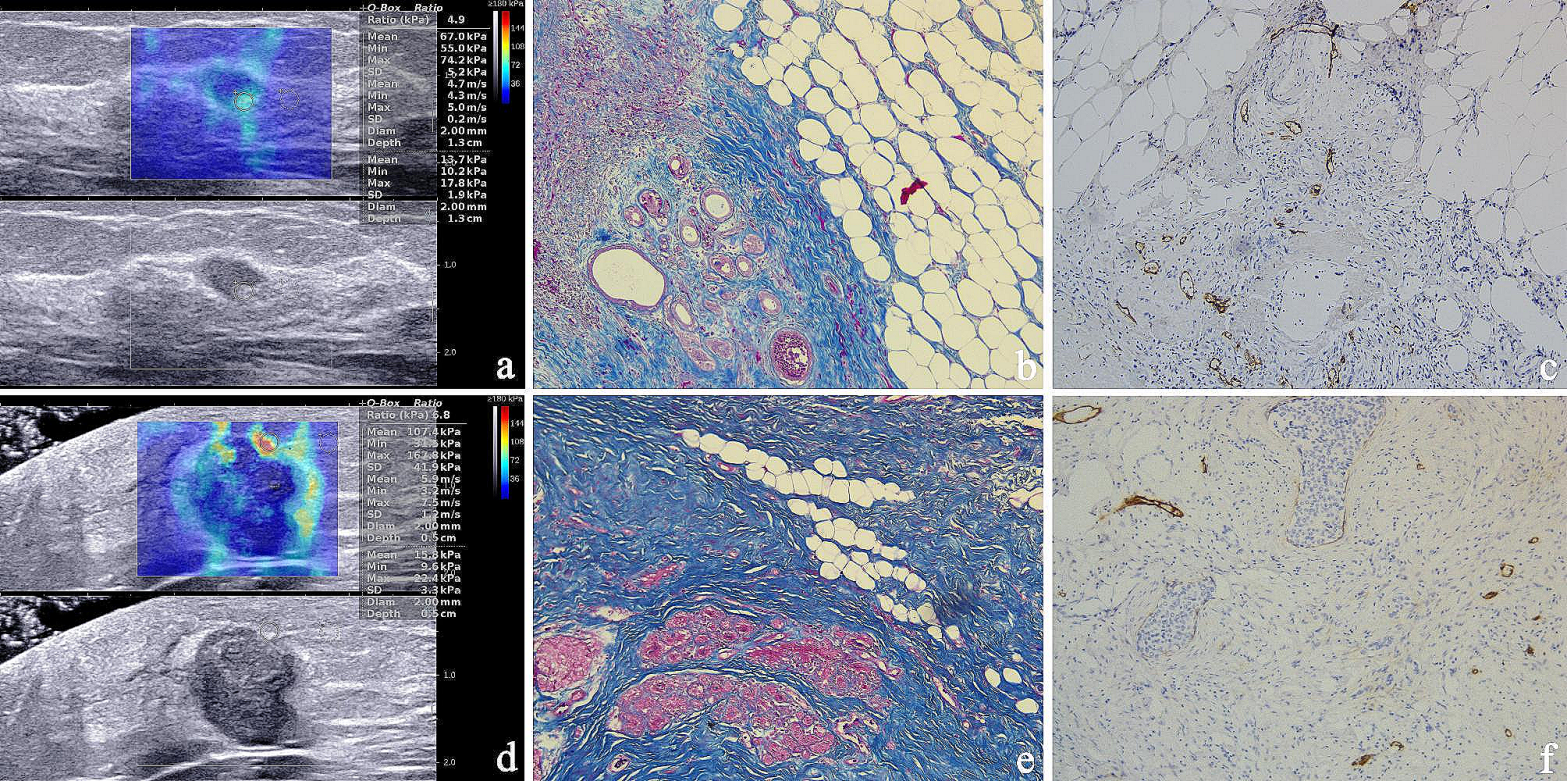
SWE parameters (a, d), the expression of collagen (b, e, x100) and MLD(c, f, x100) in LNM- (a, b, c) and LNM + patients (d, e, f). MLD microlymphatic density

A correlation analysis among SWE parameters, CVF and MLD of 97 patients with primary breast cancer was conducted (Table 6). Results showed that only CVF and Emean had a weak negative correlation (Pearson correlation coefficient analysis ρ=-0.223, *P* = 0.028, Spearman rank correlation coefficient analysis *r*=-0.204, *P* = 0.045). The correlation between CVF and Emean is shown by a scatter plot (Fig. 3). There was no correlation between the SWE parameters and MLD in primary breast cancer lesions. Figure 4 shows SWE parameters, the expression of collagen and MLD in LNM- and LNM + patients.

**Table 6:** The correlation analysis among SWE parameters, CVF and MLD

Variables 1	Variables 2	ρ	*p*-value (Pearson)	r	*p*-value (Spearman)
MLD	Emax	0.02	0.847	0.026	0.8
MLD	Emean	0.016	0.876	-0.042	0.685
MLD	Eratio	0.08	0.436	0.093	0.367
MLD	Esd	0.114	0.064	0.016	0.878
CVF	Emax	-0.15	0.143	-0.092	0.37
CVF	Emean	-0.223	0.028	-0.204	0.045
CVF	Eratio	-0.032	0.754	0.001	0.991
CVF	Esd	0.108	0.293	0.108	0.292
CVF	MLD	0.084	0.412	0.08	0.439

*MLD* microlymphatic density, *CVF* collagen volume fraction

## Discussion

The presence of LVI indicates an increased risk of metastasis in breast cancer. Some studies have shown that LVI is associated with poor prognosis. Although the vascular density of breast cancer is higher than that of LVD, tumor emboli are reported to mainly occur in lymphatic vessels [12]. Zhang et al. [13] showed that the detection rate of LVI in the LNM + group was higher than that in the LNM- group, and LVI was a valuable predictor of LNM + in breast cancer. The results of this study are consistent with the above research. In most clinical institutions, immunohistochemistry is not routinely used for preliminary screening of LVI, which limits the clinical application of LVI as a powerful predictor of breast cancer.

Studies have shown that tumor size is an independent predictor of LNM [14]. This research also confirms this viewpoint. The larger the tumor is, the larger the area of adjacent tissues invaded by cancer is, and the higher the risk of LNM is [15]. Posterior feature enhancement is related to the relative retention of transmitted ultrasound in the distal part of the mass [16]. Fluid, mucus, hemorrhage, or necrosis may usually lead to the appearance of posterior feature enhancement [17, 18]. When the cells in the tumor are loosely arranged, the fibrous interstitial component is small, or the distribution is not uniform, additionally there is liquefaction and necrosis inside the tumor, the echo behind them shows a combined pattern or even enhancement, which is considered to indicate relatively weak invasion and infiltration capacity. This study confirmed that patients in the LNM- group were more likely to exhibit posterior feature enhancement.

Most previous studies [19–23] have provided the optimal cutoff values for the diagnosis of the malignant group. Wen et al. [24] reported that there was a significant difference in SWE values between the LNM + group and the LNM- group. Our study confirmed this conclusion. However, the optimal cutoff values of Emax, Emean, and Eratio for diagnosing LNM + were 111.05 kPa, 79.80 kPa, and 6.89, respectively, which were lower values than the ones we found. This might have been due to the different probe frequencies. Although SWE has a high reproducibility within and between observers [25], SWE values vary between and within observers. The cause might also be the Q-box. We put the Q-box in the hardest part of the peritumoral lesions whereas Wen put it in the hardest of the mass. However, the diagnostic accuracy of Emax in this research was low, so we tried to combine multiple indicators which were described as the logistic regression model to improve the diagnosis of LNM more effectively.

In recent years, several studies have included correlation analyses between SWE and collagen. Xue et al. [26] found that collagen expression was significantly different in benign and malignant breast lesions. The expression of collagen was positively correlated with Emax but not with Emean. However, there was no difference in the expression of collagen between the LNM + and LNM- groups. Wang et al. [27] found that Emax was positively correlated with the collagen fiber component. Wen et al. [24] reported that the CVF of breast cancer patients with LNM + was significantly higher than that of those with LNM-, and Emax, Emean, and Eratio were positively correlated with CVF. According to shape and arrangement characteristics, Shi et al. [28] divided collagen fibers into four categories, and the results showed that collagen fibers themselves and their arrangement or integrity affected the elasticity of tissues. The results of this study showed that Emean and CVF had a weak negative correlation, which was inconsistent with the above results. This might have been due to the different inclusion criteria of patients and the different placement of the Q-box. Further studies are needed on the correlation between collagen and SWE values in breast cancer.

The role of intratumoral and peritumoral lymphatic vessels in the development of breast cancer has long been controversial. In the peritumoral stroma, there is inflammation and dilation of the lymphatic vessels, which are the main routes for cancer cell transmission. In contrast, the existence of intratumoral lymphatic vessels is unclear, and most of the intratumoral lymphatic vessels are sparse, dysfunctional and collapsed pipelines that are long and narrow or atretic, and not conducive to lymphatic drainage or cell migration [29]. Zhang et al. [13] believed that compared with intratumoral LVD, peritumoral LVD was more strongly correlated with LVI and LNM, and peritumoral lymphatic vessels were the main transmission route of breast tumor cells. In this study, the primary concern was pericancerous MLD, and it was found that the parameters of SWE in primary breast cancer were not related to MLD. In addition, there was no significant correlation between CVF and MLD. However, Cha et al. [30] found that the Emean of breast cancer was related to MLD. The controversial conclusions might have been because this study only focused on the peri-cancerous SWE value and the expression of CVF and MLD but did not pay attention to the parameters of SWE in the stiffness part of the primary breast cancer (possibly intratumor or peritumor). Therefore, an expanded sample size and further studies are needed to explore the correlation between MLD and SWE parameters intratumorally.

There are some limitations in this study. This was a single-center study, which required more samples from multiple centers to obtain accurate diagnostic reference values of SWE. Eligible patients undergoing NAC were not included in the study, which might have resulted in bias. In the research, the final pathological results of all axillary lymph nodes in patients were not available due to the non-routine performance of ALND on patients with negative sentinel lymph node biopsy, and they were presumed to be free from LNM, it perhaps made the positive lymph nodes initially missed which could cause measurement error in coding LNM status. In the study of the correlations among SWE parameters, CVF, and MLD, only peritumor parameters were evaluated, and subsequent intratumoral analysis of related parameters is still needed.

## Conclusions

In conclusion, the logistic regression model can help us to determine LNM, thus providing more imaging basis for the selection of preoperative treatment. The SWE parameter of the primary breast cancer lesion cannot reflect the peritumoral lymphangiogenesis, and we still need to find a new ultrasonic imaging method.

## Data Availability

The datasets used and/or analyzed during the current study are available from the corresponding author on reasonable request.

## Abbreviations

SWE: Shear wave elastography
LNM: Lymph node metastasis
IBC: Invasive breast cancer
CVF: Collagen volume fraction
MLD: Microlymphatic density
PPV: Positive predictive value
NPV: Negative predictive value
ALND: Axillary lymph node dissection
ROI: Region of interest
LVD: Lymphatic vessel density
ECM: Extracellular matrix
NAC: Neoadjuvant chemotherapy
BI-RADS: Breast Imaging Reporting and Data System
Emax: Maximum stiffness
Emean: Mean stiffness
Eratio: Stiffness ratio
Esd: Stiffness standard deviation
ER: Estrogen receptor
PR: Progesterone receptor
Her2: Human epidermal growth factor receptor 2
ROC: Receiver operating characteristic
LVI: Lymphovascular invasion
AUC: Area under the curve
